# Circular causality in volition

**DOI:** 10.3389/fnetp.2025.1631899

**Published:** 2025-07-16

**Authors:** Hans Liljenström

**Affiliations:** ^1^ Agora for Biosystems, Sigtuna Foundation, Sigtuna, Sweden; ^2^ Department Energy and Technology, Swedish Universitey of Agricultural Sciences (SLU), Uppsala, Sweden

**Keywords:** brain dynamics, network physiology, neural connectivity, EEG, synergetics, neurocomputational modeling, downward causation, free will

## Abstract

Conventional scientific paradigms predominantly emphasize upward causality, often overlooking or dismissing the role of downward causality. This approach is also prevalent in neuroscience, where cortical neurodynamics and higher cognitive functions are typically viewed as consequences of neuronal or even ion channel activity. Conversely, mental phenomena are generally assumed to lack causal efficacy over neural processes—an assumption that is increasingly being questioned. The causality associated with volition may be analyzed at three organizational levels: (1) neuronal interactions within cortical networks, (2) interregional dynamics between distinct brain areas, and (3) the reciprocal relationship between the nervous system and its environmental context. Across all these domains, circular rather than strictly linear causality appears to be at play. This paper examines the implications of such circular causality for volition and the longstanding problem of free will, with particular reference to insights derived from neurocomputational modeling.

## 1 Introduction

A central challenge in the study of *volition* lies in understanding causality. Volition refers to the conscious act of choosing or deciding, closely tied to intentionality, agency, and the notion of free will. *Causality*, traditionally viewed within a deterministic framework, implies that every event is necessitated by prior events according to natural laws. This raises a fundamental question: Are our actions solely initiated by neural processes in response to external stimuli, or do we, as conscious agents, possess the ability to make self-initiated decisions?

Free will, commonly defined as the capacity to make choices not strictly determined by prior causes, remains a core issue in *neurophilosophy* (Maoz and Sinnott-Armstrong, 2022). In our research, we approach this problem using neurocomputational modeling to explore the brain’s structural and functional underpinnings of volition.

We conceptualize the brain as a complex system, with activity reflected in complex neurodynamics, including oscillations and fluctuations observable in EEG recordings. These dynamics result from interactions among multiple organizational levels, from ion channels and neurons to networks, each governed by distinct time scales and regulatory mechanisms. Also input from the environment affect the brain dynamics in ways hard to control.

Complex systems typically exhibit non-linear interdependencies, thresholds, and feedback loops, which contribute to unexpected or non-intuitive behaviors. These properties demand a multiscale perspective to properly capture causal interactions (Liljenström, 2016). As noted in *cybernetics* (Wiener, 1948) and *systems neuroscience* (Érdi, 2024), feedback in goal-directed systems, such as animals and certain automatic machines and robots, makes a distinction between cause and effect difficult, since the output influences the input.

Hermann Haken’s concept of *synergetics* (Haken, 1973; Haken, 1991) further clarifies this type of complex systems, with additional concepts to describe their behavior: “An important result of synergetics consists in the insight that close to [such] instability points the system’s dynamics is governed by, in general, few quantities–the *order parameters*. Once their dynamics is determined via the *slaving principle*, the behavior of the individual parts is fixed, perhaps with exception of small stochastic fluctuations. In turn, the dynamics of the order parameters is brought about by the cooperation of the individual parts of the system. Thus, order parameters determine the behavior of the parts, while the parts determine the behavior of the order parameters. This phenomenon is called *circular causality* (Haken, 2005).

Haken applied this approach to the human brain-mind system (Haken, 1996; Haken, 2008), suggesting that “…the thoughts are order parameters and the neurons the enslaved parts. Note that in this way one would say that our thoughts are the outcome of the cooperation of myriads of neurons, but that on the other hand, the action of the neurons is enslaved by our thoughts. It should be mentioned, however, that there are in my opinion, fundamental limits to the treatment of the brain by these concepts, because qualities, such as perception of the color, feelings, etc., are not covered by this approach. There still seems to be a fundamental difference between the action of our brain, and say, that of a machine” (Haken, 1997).

In addressing volition, we aim to clarify the intricate causal relationships within brain dynamics, as determined by network physiology and connectivity. Neural activity is typically assumed to cause mental phenomena (upward causation), while the reverse—mental states influencing neural processes (downward causation)—is often neglected, with some insightful exceptions (see Murphy et al., 2009). This issue was addressed already by Szentagothai (1984), although he questioned the use of the term *causation/causality* when two different categories (mental and physical) are involved. Demonstrating downward causation, however, requires showing that changes in higher-level variables reliably affect lower-level dynamics, a condition that may be necessary, but not sufficient for substantiating free will.

Our neurocomputational models simulate various cortical regions, examining how their neurodynamics depend on structural factors such as connectivity and cell types, as well as intrinsic and external signals. We also explore how the complex neurodynamics may influence neuronal populations and relate to mental functions. This is particularly relevant for the *action–perception cycle*, which is meaning-making in animal behavior (Freeman, 1999; Freeman, 2000), a view far from the simplistic stimulus-response paradigm of traditional behaviorism (Skinner, 1974).

The action-perception cycle is at the heart of Freeman’s theory of cortical dynamics, describing an organism’s interaction with its environment through a continuous loop of action and perception in search of food or social partners. Volitional actions are typically preceded by intentions, while perceptions are shaped by attention. These processes may not be sequential but parallel, involving distinct neural hierarchies. Thus, intention and attention, as key aspects of consciousness, should be viewed as interdependent and equally vital for adaptive behavior (Liljenström, 2011; Liljenström, 2022).

In the sections that follow, we explore how circular causality manifests at three interrelated levels relevant to volition: (1) within cortical neural networks, (2) across different brain regions, and (3) in interactions between the individual and the external environment.

## 2 Interaction between cortical network neurons

Mesoscopic brain dynamics emerge from a delicate balance between excitation and inhibition, often producing oscillatory or more complex activity patterns (Freeman, 2000; Liljenström, 2012). This activity is mixed with microscopic noise from spontaneous neural firing and modulated by macroscopic processes, such as slow cortico-thalamic rhythms and neuromodulation from various brain regions. Top-down influences, like arousal, attention, or mood, further emphasize the impact of macroscopic activity on mesoscopic dynamics.

It is evident that intrinsic noise can induce various phase transitions within network dynamics, potentially influencing higher-order functions. For instance, an elevated noise level localized to a small subset of network nodes can trigger widespread spatio-temporal oscillations across model networks, thereby recruiting otherwise inactive nodes into global network activity (Liljenström, 1996; Basu and Liljenström, 2001). Notably, such noise-induced phenomena may even enhance the efficiency of the system’s information processing capabilities (Liljenström and Wu, 1995).

The complex neurodynamics of cortical networks can also exhibit chaotic behavior, with high sensitivity to initial conditions. Even small variations in firing patterns can lead to drastically different network outputs. Both experimental research (Skarda and Freeman, 1987; Freeman, 2000) and computational models (Liljenström, 1995; Hassannejad Nazir et al., 2023) indicate that chaotic (intentional) states may emerge from this dynamics and later stabilize into coherent percepts, potentially linked to decision-making.

**FIGURE 1:**
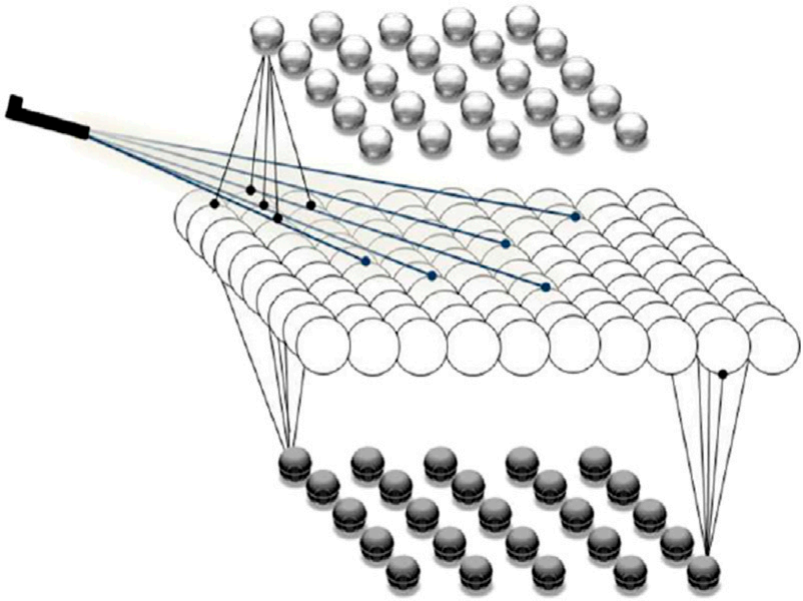
Model of the cortical network structures used in our study. The upper and lower layers are each composed of 25 inhibitory nodes, corresponding to inhibitory interneurons, and the middle layer is composed of 100 excitatory nodes, corresponding to populations of pyramidal cells. External inputs stimulate a subset of the excitatory nodes, and all the excitatory nodes may excite the inhibitory ones. The model simulates a 10 × 10 mm square of each cortical structure.

Our simulations show how microscopic events at the level of single neurons, or neuronal populations, can shape mesoscopic dynamics, which in turn relate to macroscopic cognitive functions. The modeled cortical structure includes one excitatory layer flanked by two inhibitory layers, representing pyramidal cells and two types of inhibitory interneurons, respectively. Excitatory network nodes are recurrently connected, and are also exciting the inhibitory nodes, while the inhibitory nodes are not interconnected. A bidirectional excitatory-inhibitory balance is maintained through distinct feedback loops (see Figure 1), enabling rich neurodynamic behavior, especially oscillations with structure-specific amplitudes and frequencies. Neural node activity represents the mean membrane potential of a neuronal population, producing graded outputs.

Network dynamics depend mainly on synaptic weight strengths and an order parameter, ***Q***, reflecting neuromodulator levels, or motivational states. A higher ***Q*** indicates stronger motivation toward a particular choice. Motivation arises from both internal experiences and external factors, and is modelled either as a constant or with varying values (For more details, see Hassannejad Nazir et al., 2023).

## 3 Interactions between brain areas

*Decision making* (DM), as an essential part of volition, is often less rational than we assume, as shown by Kahneman (2011) and Kahneman and Tvserky (1979). According to their dual-system theory, DM arises from the interplay between two systems: a fast, intuitive, emotional one (System I), and a slower, deliberate, rational one (System II) (Kahneman, 2011). System I operates implicitly and bottom-up, while System II functions explicitly and top-down.

To investigate how different brain regions interact during DM and volition, we developed neurocomputational models that simulate various experimental scenarios (Hassannejad Nazir and Liljenström, 2015; Hassannejad Nazir and Liljenström, 2016). While several brain areas may be involved, we focus on the lateral prefrontal cortex (LPFC), orbitofrontal cortex (OFC), amygdala, and anterior cingulate cortex (ACC) — key structures in both DM and *intentional control* (IC) (Hassannejad Nazir et al., 2023).

**FIGURE 2:**
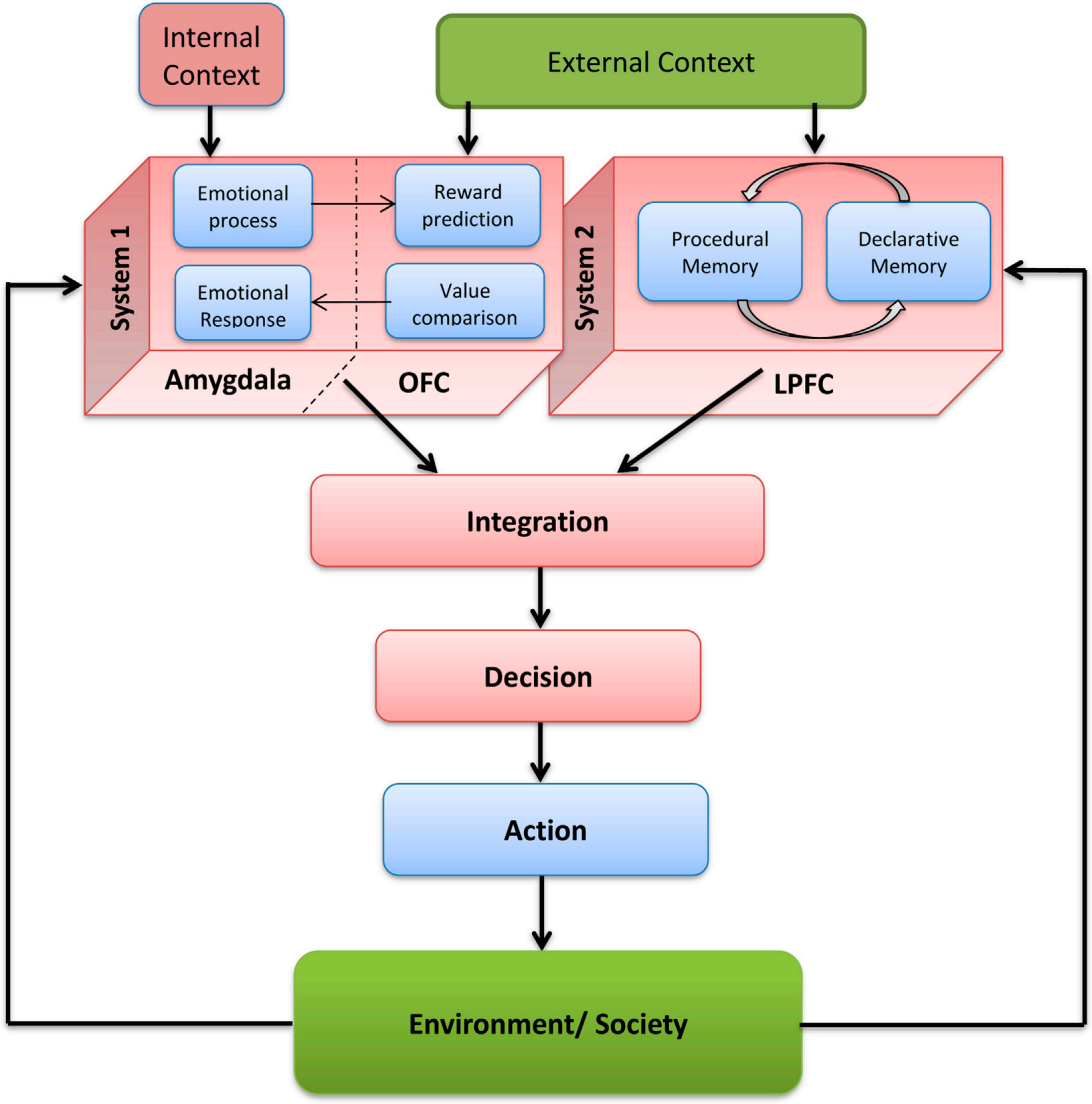
Schematic flow chart of the subsystems and information flow in the modelled decision making process, including feedback within and without the brain-mind system (Adopted from Hassannejad Nazir and Liljenström, 2015).

Our DM model (Figure 2) incorporates Kahneman’s dual-system framework. It uses a value function to evaluate potential outcomes based on the expected reward, delay, and probability, favoring actions with the highest overall value. Learning from experience requires identifying the specific actions that led to particular outcomes. In the model, different decision options are represented by dynamic *cell assemblies* oscillating in the gamma range (30–80 Hz), corresponding to EEG signals from the relevant brain regions.

Numerous internal and external factors influence DM, including desires, risks, and environmental uncertainty. For example, a large expected reward might drive behavior despite high costs, while uncertainty can lead to increased risk-taking and exploratory choices. In contrast, predictable situations support long-term planning.

Time is a key parameter here: in intertemporal DM, future rewards are often undervalued—a tendency that leads to prioritizing immediate over delayed benefits, although this generally leads to a net loss (Doya, 2008). This bias may explain why urgent issues like climate change are often neglected, because decisions have to be made long before the effects are noticed. Emotionally driven short-term rewards likely involve limbic regions, while long-term planning is primarily managed by rational, neocortical areas.

Effective DM is adaptive, influenced by individual experiences, preferences, attitudes, and social context. While attitudes are generally relatively stable, they can evolve at a longer time-scale through learning and social interaction. Our models also consider these influences (see next section and Hassannejad Nazir and Liljenström, 2015; Hassannejad Nazir and Liljenström, 2016).

Decisions to act are closely linked to the readiness potential (RP) observed in Libet-type experiments (Libet et al., 1982; Libet et al., 1983; Soon et al., 2008; Haynes, 2011). Although the significance of RP for volition has been debated (Schurger et al., 2012; Schurger, 2018), current consensus holds that voluntary actions are preceded by RP (Haggard and Eimer, 1999; Travers et al., 2020; Schultze-Kraft et al., 2020).

We hypothesize that decisions arise from competition between System I (Amygdala/OFC) and System II (LPFC) dynamic cell assemblies. The “winning” signal proceeds to the supplementary motor area (SMA), initiating motor output. Our IC model investigates how RP might originate from earlier activity in LPFC, linked to rational decision-making (Hassannejad Nazir et al., 2023).

The contribution of ACC to both rational and emotional aspects of human behavior makes it a hub for cortico-cortical and cortico-limbic connections (Hayashi, 2006). Hence, ACC can be regarded as a hub for social valuation. The afferent and efferent fibers connecting different neural structures, including LPFC and OFC to ACC, facilitate the flow of social information between these structures.

Our neurocomputational models illustrate the complex, dynamic interactions between brain areas involved in volition, where circular causality plays a central role. A key challenge is identifying where intentions originate and how they relate to earlier and subsequent decisions and actions. While models cannot fully explain brain processes, they can offer valuable insights.

Volitional DM involves selecting among alternatives through conscious control. This process may be underpinned by complex, possibly chaotic, neurodynamics that eventually stabilize into more ordered, oscillatory activity associated with decision resolution. Our model-generated EEG-like signals help explore such oscillatory shifts, such as the transition from beta to gamma rhythms in the prefrontal cortex.

We have also simulated the evolution of neural attractor states and shown how intention may emerge through hierarchical processing of external and internal stimuli, as well as goal retrieval. These attractor states are modulated through feedback loops at multiple levels (Hassannejad Nazir et al., 2023).

## 4 Interaction between individual and environment

The choice of action, deciding among several options, is shaped by a range of internal and external factors. Whether consciously or unconsciously, we are influenced by others, both past and present. While personal experience often drives our behavior, social learning also plays a key role (Campbell-Meiklejohn et al., 2010). In particular, decision making is closely tied to learning (Gold and Shadlen, 2007; Lee et al., 2012), where our choices are influenced by cultural, societal, and interpersonal interactions.

For instance, to encourage environmentally responsible behavior, authorities may offer incentives like subsidies for installing solar panels, buying electric cars, or organize recycling systems. A municipality and bus company might jointly offer free monthly tickets to commuters who leave their cars at home and instead take the bus to work. Often, early adopters inspire others, neighbors or coworkers, to follow suit, and over time, such behaviors become community norms, even after the incentives are removed (Liljenström and Svedin, 2016).

Positive or negative behaviors observed in others tend to impact our own decisions. Observing others (including figures of authority) can alter trust and inspire behavioral shifts (Bandura, 1977). This process, known as *observational learning* (Ramsey et al., 2021), relies on associating observed actions with outcomes, generating prediction error (PE) signals that drive learning.

The link between learning and goal-directed behavior extends to *trust*. As a form of social capital, trust is shaped by expectations and prediction accuracy (Rompf, 2015). Trust influences how we interpret others’ actions and outcomes and, in turn, how we learn and decide. Greater trust improves learning efficiency and decision quality (Frith and Singer, 2008).

Interpersonal trust is closely tied to societal influence. Observing trusted individuals or authorities can shift one’s behavior, depending on the perceived reliability and consistency of their actions. In essence, observing others forms a foundation for learning and trust-building. As individuals interpret action-outcome relationships, predictability fosters trust, shaping both emotional and rational evaluations. Trust and observational learning thus emerge as key social forces that shape behavior and attitudes.

In the context of volition, it is especially important to trace the neural pathways involved, from intention formation to decision-making and action execution. Also in complex social systems, cause and effect are often difficult to isolate due to the interconnected nature of our environments. Even in controlled experiments on volition, such as Libet-type paradigms, so-called self-initiated actions may be subtly influenced by external cues (including the experimenter). This again suggests the relevance of circular causality in understanding volitional behavior.

## 5 Discussion

This paper has examined the causal dynamics of volition within the brain–mind system, highlighting the inherent difficulty of identifying linear cause–effect relationships in the action-perception cycle. When our self-initiated actions are based on conscious decisions, we may feel we are acting out of free will, but it is not clear how free we really are from external influences. Our decisions may be based on a number of circumstances, knowledge, memories, attitudes, preferences, feelings, impressions, etc.,–all of which may have come to us from the environment or other individuals.

While the concept of freedom is much debated in philosophy it may be confusing when approaching volition scientifically. Hence, it can be argued that *conscious will* is a better concept than free will. However, drawing on both neuroscience and computational modeling, we argue that the traditional debate on free (conscious) will, whether it exists or is an illusion, is misframed (Wegner, 2002; Sapolsky, 2023). Instead, our findings support a model of circular causality, where mental and neural processes mutually influence each other.

As we have seen from the examples above, cause and effect are difficult to separate in complex networks of interconnected entities at different levels of organization. In such complex, feedback-driven systems, clear causal chains are futile to look for. Neural activity may precede, follow, or occur simultaneously with mental events, and both are shaped by internal and external factors: genetic, physiological, environmental, and social. This interconnected web of influences renders human decisions highly unpredictable based on neural signals, with outcomes potentially shaped by both upward and downward causation.

Ultimately, the complexity of brain organization and the embeddedness of the individual in a dynamic social and physical environment challenge simplistic interpretations of volition. The study of free (conscious) will must account for the continuous interplay between neural systems, subjective experience, and environmental context, highlighting the need for integrative approaches that go beyond reductionist models. Our own neurocomputational modeling supports the view that decisions may result from interactions within and without the brain-mind system, with an intentional process that gradually becomes more and more conscious (see Hassannejad Nazir et al., 2023, for further results).

Contrary to the dominant view that consciousness lacks causal power and that conscious will is merely an illusion, I argue that the concept of circular causality provides a compelling framework for understanding the experience of agency. To date, neurophysiological experiments, such as those by Libet and others, have not offered conclusive evidence against the existence of conscious will (Liljenström, 2022). Elsewhere, I have also argued that the laws of physics may not be fully applicable to the brain–mind system or agency (Liljenström, 2025).

Notably, even conclusions drawn from one of the leading theories of consciousness, Integrated Information Theory (IIT), suggest that consciousness may indeed possess causal powers (Tononi et al., 2023). This perspective resonates with Hermann Haken’s assertion that “Mind and matter condition each other, or in other words, mind and matter are two sides of the same coin. This is my point of view, but it is not new … it is that of Spinoza” (Haken, 1996).

## Data Availability

The original contributions presented in the study are included in the article/supplementary material, further inquiries can be directed to the corresponding author.
